# Longitudinal, prospective cohort study of social relationships and self-rated health in the Atherosclerosis Risk in Communities (ARIC) Study cohort and ARIC/Jackson Heart Study (JHS) shared cohort

**DOI:** 10.1371/journal.pone.0326196

**Published:** 2025-06-13

**Authors:** Kennedy M. Peter-Marske, Annie Green Howard, Kelly R. Evenson, Sara Jones Berkeley, Joanna Maselko, Mario Sims, Anna Kucharska-Newton, Kevin J. Sullivan, Wayne D. Rosamond

**Affiliations:** 1 Department of Epidemiology, Gillings School of Global Public Health, University of North Carolina at Chapel Hill, Chapel Hill, North Carolina, United States of America; 2 Department of Biostatistics, Gillings School of Global Public Health, University of North Carolina at Chapel Hill, Chapel Hill, North Carolina, United States of America; 3 Carolina Population Center, University of North Carolina at Chapel Hill, Chapel Hill, North Carolina, United States of America; 4 Department of Social Medicine, Population and Public Health, School of Medicine University of California at Riverside, Riverside, California, United States of America; 5 Department of Medicine, The MIND Center, University of Mississippi Medical Center, Jackson, Mississippi, United States of America; Northern Arizona University, UNITED STATES OF AMERICA

## Abstract

**Introduction:**

Social isolation and low social support are associated with low self-rated health (SRH) cross-sectionally, but few studies have assessed longitudinal associations.

**Aims:**

Assess the associations of isolation, support, and different types of support with SRH trajectories over 28 years. Examine 10-year changes in isolation and support and their associations with 18-year SRH trajectories.

**Methods:**

The Atherosclerosis Risk in Communities (ARIC) Study and the Jackson Heart Study (JHS) are population-based prospective cohort studies with some shared participants. We included 10,855 ARIC participants (56% women, 24% Black, mean age 57 (standard deviation: 6) years) with one measure of isolation and support in ARIC (1990–1992), and a subset of 911 ARIC/JHS shared cohort participants with these measures again in JHS (2000–2004). Isolation was measured using the 10-item Lubben Social Network Scale in ARIC, and with 3 questions from the Berkman Social Network Index in JHS. Support was measured using the 16-item Interpersonal Support Evaluation List in both studies. SRH was measured annually and scored from 0–100. We used linear mixed effects models adjusted for confounders to assess these associations.

**Results:**

In ARIC, high isolation was associated with lower SRH both at baseline and over follow-up, with SRH decreasing at a slightly greater rate for those with high isolation compared to low. High support was associated with greater SRH over 28 years compared to those with low support, but the rate of decline in SRH was similar. On average, over 10 years, support was stable and isolation increased in ARIC/JHS. Although confidence intervals were wide, 10-year maintenance of high/moderate support and increases in support were associated with greater SRH over time compared to decreases in support and stable low support.

**Conclusion:**

Low isolation and high support at baseline and over 10 years may be positively associated with longitudinal SRH.

## Introduction

The quantity and quality of social relationships impact health and well-being. High social isolation and low social support are associated with greater risk of all-cause mortality [1], and poor mental and physical health outcomes [1–7]. The Coronavirus-19 pandemic has created a renewed urgency to understand how social relationships and changes in social relationships affect health over time as the prevalence of social isolation, loneliness, and mental health conditions have increased since 2020 [8–11]. While much is yet to be learned about the downstream effects of pandemic-related changes in social relationships on long-term general health, leveraging existing data may yield key insights into these associations.

Self-rated health (SRH) is a simple, single item measure of a complex construct that combines perceived physical, mental, and social well-being [12]. SRH is associated with many clinical outcomes such as mortality, incident stroke, and healthcare utilization [12]. More favorable measures of social relationships have been cross-sectionally related to better SRH, but few studies have investigated the associations of mid-life social relationship measures with longitudinal changes in SRH [13,14].

Evidence is also needed to assess the stability of social isolation and social support in mid-life and whether changes in these measures are associated with long-term perceptions of health. Social relationships may change in the presence of life events that alter social circles, such as marriage, divorce, death of a partner, relocation, and retirement [15,16]. Social support and social isolation may be modifiable through interventions such as cognitive behavioral therapy [15] and peer support programs [17,18]. By investigating which aspects of social relationships and changes in social relationships are most associated with maintenance of better SRH, we may be able to identify specific aspects to target for future interventions.

Therefore, we examined how social isolation, social support, and different types of social support (appraisal, belonging, self-esteem, and tangible support) at mid-life were associated with annual repeated measures of SRH over 28 years in the Atherosclerosis Risk in Communities (ARIC) study. We additionally assessed how 10-year changes in these social relationship measures among participants enrolled in the ARIC and Jackson Heart Studies (JHS) were associated with longitudinal changes in SRH over 18 years. We hypothesized that participants with high social isolation, low social support, and negative changes in these measures would have poorer SRH at baseline and a greater rate in decline of SRH over time than participants with better social relationship measures.

## Methods

### Study design and population

Middle-aged (45–64 years), predominantly White and Black men and women were followed as part of the ARIC study, a population-based prospective cohort study that began in 1987–1989 (Visit 1). Participants were recruited from four communities in the United States (Forsyth County, NC; Jackson, MS; suburbs of Minneapolis, MN; and Washington County, MD), amounting to 15,792 participants who attended Visit 1 (participation rate of 60%) [19]. These participants were invited to attend subsequent in-person visits and annual follow-up telephone calls; semi-annual follow-up calls began in 2012. Further details of the ARIC study have been published elsewhere [20]. The ARIC study was approved by the Institutional Review Boards at the study centers and coordinating center: Wake Forest University, University of Mississippi Medical Center, University of Minnesota, Johns Hopkins University, and the University of North Carolina at Chapel Hill. Written informed consent was obtained from all participants. Data were originally accessed for this analysis in January 2023, and can be requested through the Biologic Specimen and Data Repository Information Coordinating Center (BioLINCC) website (https://biolincc.nhlbi.nih.gov/studies/aric/) after registering with the site.

Our analysis population included 14,348 participants who attended Visit 2 from 1990–1992, as this was the first assessment of social relationships. We excluded participants who self-reported a race other than Black or White (n = 42) and Black participants from the suburbs of Minneapolis, MN or Washington County, MD (n = 49) due to small sample sizes. We further excluded participants with incomplete measures of social isolation or social support (n = 1,074). For main analyses, we excluded participants with prevalent cancer (n = 887), myocardial infarction (n = 711), coronary heart disease (n = 180), stroke (n = 151), or heart failure (n = 399) at Visit 2 to minimize potential reverse causation, as the relationship between social relationships and health is suggested to be bi-directional. Those with poor social relationships may experience poorer health in the future, and serious health conditions may alter social relationships either by hindering the ability to participate socially or by creating patient-caregiver relationships that may not have existed prior to disease onset [21]. This resulted in a final analytic sample of 10,855 participants for whom there are a maximum of 28 annual follow-up phone calls through 2020.

The Jackson Heart Study (JHS), a prospective cohort study of Black men and women from the tri-county area of the Jackson, MS metropolitan area, began in 2000–2004 [22]. Of the total 5,306 JHS participants, 1,625 individuals were participants of the ARIC study (participation rate of 39% among eligible ARIC participants); our analysis utilized data only from JHS participants who were also a part of the ARIC cohort [22]. Additional details of the JHS have been previously published [23,24]. Co-participants of the ARIC study and JHS have two measures of social relationships approximately 10 years apart: one at ARIC Visit 2 (baseline), and one at JHS Visit 1 (2000–2004). All ARIC/JHS co-participants gave written informed consent for the JHS, and protocols for the JHS were approved by the Institutional Review Board at the University of Mississippi Medical Center. Data from JHS can be accessed by request. For analyses of 10-year changes in social relationships among the 1,625 ARIC/JHS cohort members, we excluded participants with missing social relationship measures at either time of measurement (n = 600). We further excluded those with the following prevalent conditions at baseline: cancer (n = 20), myocardial infarction (n = 36), coronary heart disease (n = 5), stroke (n = 11), and heart failure (n = 53). This resulted in a final sample size for analyses of 911 ARIC/JHS co-participants for whom there are a maximum of 18 annual follow-up phone calls following the second social relationship measurement through 2020.

### Social relationships

Social isolation measures relationship quantity by assessing the frequency and number of social contacts, along with involvement in social networks [25]. The Lubben Social Network Scale (LSNS) was designed as a screening tool to identify individuals at risk of social isolation and was implemented at baseline. The LSNS is a well validated [26] questionnaire with high internal reliability, and is based on the full Berkman-Syme Social Network Index (BSNI) [27]. The LSNS measures the self-reported availability of social contacts with family, friends, and peers, along with network size. It uses 10 questions with a 0–5 rating scale. Total scores ranging from 0–50 have been categorized into levels of risk for social isolation: socially isolated (≤20), high risk (21–25), moderate risk (26–30), and low risk (≥31) [28].

Frequency and number of social contacts were assessed using three items adapted from the BSNI for the second measure of social isolation [29]. Both the LSNS and BSNI measure involvement in relationships [25]; therefore, we created a version of 4 LSNS questions most similar to those of the BSNI to assess changes in social isolation. Details of the three BSNI questions used in the JHS and the modified LSNS questions and scoring can be found in S1 Table. For the BSNI social isolation score, the threshold at which participants were considered socially isolated/high risk for social isolation was determined by comparing the established categories for risk of social isolation from the LSNS to the median score of the 0–12 point BSNI-derived measure within each category of isolation. The median score of the BSNI measure among those who were socially isolated at baseline was 5; therefore the 5.9% of participants who had a BSNI score <5 were categorized as socially isolated/at-risk high risk for isolation at baseline. This same cut point was used for BSNI scores at the second time of social relationship measurement.

Social support is a measure of relationship quality, including the types and purposes of perceived availability of social support [1,30,31]. ARIC and JHS both used the 16-item Interpersonal Support Evaluation List (ISEL) to measure total perceived social support. The ISEL uses questions from 4 subscales of the 40-item ISEL: appraisal, belonging, self-esteem, and tangible. Appraisal support refers to aid given to help explain, understand, and cope with events, and can also include referrals to other social resources [32], whereas belonging support includes social companionship and emotional support [32]. Self-esteem support is defined by social resources that provide feelings of acceptance and personal value to the recipient [32], and positive feelings of self-worth compared to others [33]. Tangible support refers to resources such as monetary assistance, material gifts, and services provided by social contacts [32]. Every question on the 16-item ISEL corresponds to one of these subtypes; scores for each specific type were calculated based on the relevant questions and ranged from 0–12. Total possible scores for the ISEL range from 0–48, with higher values indicating greater perceived social support. There are no standard categories used for the ISEL-16 for levels of social support, or for its subscales. A previous ARIC study reported a moderate correlation between the ISEL and LSNS (r = 0.45) in 14,257 participants [34].

We assessed the best specification of social support comparing a variety of different variable specifications (linear, quadratic, quantiles, etc.) using likelihood ratio tests (two-sided p-values, α < 0.05), Akaike Information Criteria (AIC), and data visualization. Based on these factors and interpretability, ISEL scores were categorized into three levels: the bottom 10^th^ percentile (≤ 29), the 11^th^-50^th^ percentile (30–38), and above the 50^th^ percentile (≥ 39), due to the non-linear association that occurred at the bottom range of social support values. We refer to these categories as low, moderate, and high social support respectively. Types of social support (appraisal, belonging, self-esteem, and tangible) were categorized using quintiles due to the limited range of values: the bottom quintile (low), quintiles 2 and 3 (moderate), and quintiles 4 and 5 (high).

Changes in social isolation and social support were assessed as an *a priori* 4 level categorical variable for analyses. For social isolation, these categories included 1) 10-year stable high risk for isolation (isolated/high risk to isolated/high risk), 2) increase in isolation (low/moderate risk to isolated/high risk), 3) decrease in isolation (isolated/high risk to low/moderate risk), and 4) stable low (low/moderate) risk for social isolation. Change in social support categories based on the cut-points outlined for low, moderate, and high social support included 1) stable low social support, 2) decrease in social support (high/moderate to low), 3) increase in social support (low to high/moderate), and 4) stable high (high/moderate) social support.

### Self-rated health

SRH has been widely validated and shown to be reproducible and reliable in many different populations [35]. SRH was measured during annual phone follow-up beginning with the first interview following the first study visit. The SRH question asks: “Over the past year, compared to other people your age, would you say that your health has been excellent, good, fair or poor?” Although a 5-item metric is commonly used to measure SRH, many variations of the SRH metric in terms of wording and number of response options consistently indicated that more favorable general health is associated with lower all-cause mortality [12]. We used a transformation of SRH categories into a semi-continuous measure which allows for deaths to be represented in analyses, as recommended for longitudinal assessment of SRH [36]. Diehr and Patrick previously calculated the probability (0–100) of being healthy (good, very good, or excellent SRH), 2 years from the present SRH measurement, conditional on the present SRH response. The probabilities correspond to the SRH categories as follows: excellent = 95, good = 80, fair = 30, poor = 15, death = 0 [37]. These probability values were then assigned to each SRH response or death accordingly at each time point, as has been done in a previous analysis of SRH over time in ARIC [38].

### Covariates

Age (continuous), self-reported sex (male or female), self-reported race (Black or White), self-reported years of education (0–12 years, GED, vocational school, years of undergraduate school, and graduate/professional school), self-reported annual household income (≤$25,000; $25,000 - $49,999; or ≥$50,000), self-reported employment status (homemaker, employed, unemployed, or retired), and use of mental health-related medications such as those used for anxiety, bipolar disorder, and depression (yes or no) were assessed at ARIC Visit 1. Age at baseline (ARIC Visit 2) was calculated based on age at Visit 1. The following covariates were assessed at baseline: self-reported marital status (married or not married), self-reported living arrangement (with spouse, with non-spouse, or alone), mental health medication use (yes or no), and vital exhaustion score, a measure that incorporates questions about depressive symptoms. Race and study center are highly correlated in the ARIC cohort; therefore, the variable race-center, a 5-level categorical classification combining field center and race (MN/White, MD/White, NC/White, NC/Black, MS/Black), was developed. Time between the two social relationship measurements was calculated in years for participants who were a part of both the ARIC study and the JHS.

### Statistical analysis

We compared the sociodemographic characteristics, social relationship traits, and cardiovascular risk factors at baseline of the entire ARIC cohort and the ARIC/JHS shared cohort. We also compared sociodemographic characteristics, social relationship traits, health behaviors, the prevalence of cardiovascular risk factors and mental health markers, and disease status at baseline and the time of second social relationship measurement by class of 10-year changes in social isolation and social support among ARIC/JHS shared cohort participants.

Confounders of the social relationship and longitudinal SRH relationship were identified through review of prior literature and the use of directed acyclic graphs. Models assessing 28-year and 18-year SRH trajectories were both adjusted for age, sex, education, annual household income, occupational status, and mental health related medications at ARIC Visit 1. Models assessing 28-year trajectories of SRH were additionally adjusted for race-center, while models assessing 18-year trajectories of SRH were additionally adjusted for the time between social relationship measures. Different specifications of continuous variables were assessed, including linear, quadratic, and categorical, using the likelihood ratio test and the AIC to determine the best fit. Based on these analyses, quadratic terms were included for age and education in 28-year analyses. All other continuous variables were modeled as continuous linear variables.

Social isolation and social support were assessed separately as exposures. We used linear mixed effects models adjusted for confounders to estimate the associations of social relationship measures at mid-life, specifically social isolation and social support data only from baseline, with SRH over 28 years. We also used linear mixed effects models to assess the associations of 10-year changes in social relationships, as defined by a 4 level categorical variable, with SRH over the subsequent 18 years. Linear mixed effects models accounted for repeated measures of SRH and used maximum likelihood estimation, included both a random intercept and slope, and assumed unstructured variance-covariance matrix for the random effects. Time was defined as continuous years since baseline for models assessing social relationships measured once, and as continuous years since the second social relationship measurement for models assessing changes in social relationships. Based on model fit statistics and visual inspection of graphs, we chose to represent time over 28 years as linear spline terms with knots at 7, 14, and 21 years, and time over 18 years with as linear spline terms with knots at 6 and 12 years. This allowed the slope of the line estimating SRH over time to change at each of these knots.

We included an interaction term between each of the social relationship exposures and time to allow for the rates of change in SRH over time to vary by level of the exposure, as was supported by fit statistics. We additionally included interaction terms between time and the following covariates, as indicated by fit statistics: sex and race-center. We investigated whether the association between social relationships and SRH over 28 years was modified by sex, race (while adjusting for center), and marital status at baseline by including three-way interaction terms between the modifier, the exposure, and time. We also investigated whether the associations of social support with longitudinal SRH were modified by social isolation, to determine whether it is important to have low social isolation even at high levels of social support. Due to the large sample size, we assessed whether the rates of change in SRH varied over time, and effect measure modification using the likelihood ratio test for three-way interaction terms, using the likelihood ratio test (p-values < 0.05 were indicative of differential rates of change or potential effect measure modification) and by visual inspection of the associations. Data were analyzed using SAS 9.4 (SAS Institute Inc).

### Sensitivity analyses

We conducted five sensitivity analyses. Non-response to annual follow-up phone calls over time ranged from <1% at year 1 to almost 26% at year 28. Participants with missing SRH data at year 28 were more commonly from Jackson, MS, Black, had slightly fewer years of education, taking mental health-related medications, and had slightly worse cardiovascular disease risk factors (hypertension, total cholesterol, and LDL cholesterol) at baseline (S2 Table). Results from linear mixed effects models are unbiased when data are missing conditional on included model covariates. We therefore conducted a sensitivity analysis further adjusting for baseline health status markers (hypertension, diabetes, total cholesterol, body mass index, and cognitive function) and other baseline variables that may be related to subsequent non-response (smoking status, alcohol use, frequency of physical exams, and health insurance status), in addition to covariates included in main models.

Second, we assessed the impact of prevalent cardiovascular disease and cancer on our results by repeating analyses including these participants rather than excluding these, as social relationship measures may be impacted by prevalent disease. Third, we adjusted social support models for social isolation, as social support is commonly thought of as a mediator of the social isolation and health relationship, to determine whether there was any remaining association between social support and SRH after adjustment. Fourth, because mental health markers measured coincident with social relationships may be mediators of the social relationship and SRH association, we further adjusted for mental health medications and vital exhaustion measured at baseline, and for depressive symptoms and stress measured at the time of the second social relationship measurement for models assessing changes in social relationships.

Fifth and finally, we assessed the impact of different change in social isolation scoring methods on the main analyses. Item scores were dichotomized (0–2 friends/family, > 2 friends/family) and summed for a total potential score range of 0–3 points. An overall score of 0–1 was considered high social isolation, while 2–3 was considered low. This was then assessed as categories of 10-year change in social isolation: stable high isolation, decrease in isolation, increase in isolation, and stable low isolation.

## Results

### ARIC participant characteristics

Among ARIC participants (mean age of 56.6 (standard deviation (SD): 5.7) years at baseline, 56% female, and 24% Black), 1.2% of participants were socially isolated, and 4.3% of participants were categorized as high risk for social isolation (Table 1). The median (25^th^, 75^th^ percentile) overall social support was 38 (33, 42). Tangible social support had the highest score of social support subtypes and self-esteem support had the lowest score.

**Table 1:** Sociodemographic and clinical characteristics of the ARIC cohort and ARIC/JHS shared cohort at Visit 2 (1990-1992).

	N (%) or mean ± SD or median [25^th^ %, 75^th^ %]	
	ARIC N = 10,855	ARIC/JHS N = 911
ARIC field center		
Forsyth County, NC	2903 (26.7)	0 (0.0)
Jackson, MS	2343 (21.6)	911 (100.0)
Minneapolis, MN	3037 (28.0)	0 (0.0)
Washington County, MD	2572 (23.7)	0 (0.0)
Age, years	56.6 ± 5.7	55.0 ± 5.4
Females	6144 (56.0)	606 (66.5)
Black participants	2624 (24.2)	911 (100.0)
Education, years	14.6 ± 4.3	15.0 ± 5.0
Employment status		
Homemaker	1274 (11.8)	63 (7.0)
Employed	7900 (72.9)	766 (84.6)
Unemployed	225 (2.1)	16 (1.8)
Retired	1434 (13.2)	60 (6.6)
*Missing*	22	6
Household income		
Under $25,000	3362 (33.0)	473 (58.6)
$25,000 - $49,999	4000 (39.2)	252 (31.2)
Over $50,000	2831 (27.8)	82 (10.2)
*Missing*	662	104
Married	8727 (82.6)	600 (71.1)
Social isolation		
Isolated (8–20)	134 (1.2)	22 (2.3)
High risk for isolation (21–25)	464 (4.3)	46 (4.8)
Moderate risk for isolation (26–30)	1447 (13.3)	140 (14.7)
Low risk for isolation (31–50)	8810 (81.2)	743 (78.1)
Social support^*^		
Overall	38 (33, 42)	39 (34, 43)
Appraisal social support	10 (8, 11)	10 (9, 12)
Belonging social support	10 (8, 11)	10 (8, 11)
Self-esteem social support	8 (7, 9)	9 (7, 10)
Tangible social support	11 (9, 12)	11 (9, 12)
Depression/anxiety medication use (Visit 1)	933 (8.6)	86 (9.4)
Depression/anxiety medication use (Visit 2)	963 (8.9)	78 (8.6)
Vital exhaustion score	9.6 ± 8.2	10.6 ± 8.3
Hypertension	167 (28.0)	360 (39.7)
Total cholesterol, mg/dL	207 ± 40	209 ± 40
LDL cholesterol, mg/dL	131 ± 37	133 ± 39
High cholesterol medication use	38 (6.4)	28 (3.1)
Diabetes	65 (10.9)	121 (13.5)
Body mass index, kg/m^2^	27.3 ± 5.3	29.8 ± 5.7

ARIC: Atherosclerosis Risk in Communities Study; JHS: Jackson Heart Study; N: number; SD: standard deviation; mg: milligrams; dL: deciliter; kg: kilograms; m: meter

*Social support variables presented as median [IQR] rather than mean (SD) due to skewed distribution

### Social relationships and self-rated health (ARIC)

**Fig 1:**
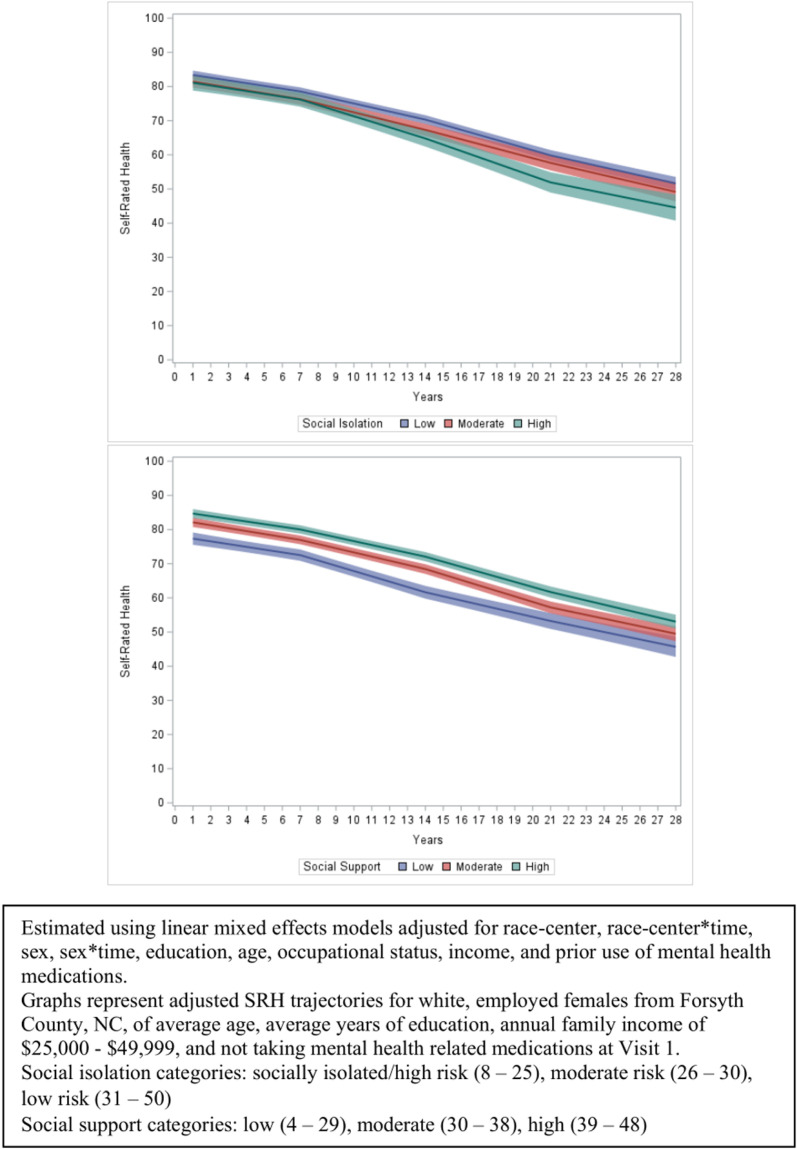
Associations of social isolation and social support with 28-year trajectories of self-rated health among Black and White middle-aged Americans in the Atherosclerosis Risk in Communities (ARIC) Study, with follow-up beginning 1990-1992.

Estimated, adjusted SRH decreased over time: SRH dropped from 83 (95% confidence interval (CI): 82, 84) to 51 (95% CI: 49, 53) over the 28 years of follow-up. High risk for social isolation was associated with lower estimated SRH over follow-up and a slightly greater rate of decline in SRH compared to those with low risk for social isolation (Fig 1). Those in the moderate risk for social isolation category consistently had an SRH trajectory between that of high and low risk for social isolation. Participants with high risk for social isolation had a slightly lower estimated SRH at the beginning of follow-up (81; 95% CI: 79, 83) compared to those with low risk for social isolation (83; 95% CI: 82, 85). By year 28, estimated SRH dropped to 45 (95% CI: 41, 48) and 52 (95% CI: 50, 54) for high risk for social isolation and low risk for social isolation respectively. Although we acknowledge that missing data may impact later years of follow-up, increasing separation in estimated SRH by social isolation categories was observed in earlier years as well: estimated SRH at year 14 for high risk for social isolation was 65 (95% CI: 62, 67) while that for low risk for social isolation was 70 (95% CI: 69, 72).

Having high overall social support was associated with greater longitudinal SRH over the course of the 28 years of follow-up compared to those with low overall social support, but rate of decline in SRH was not differential with regards to social support (Fig 1). The trajectory of SRH for moderate social support was between that of high and low social support. High overall social support began with a greater estimated SRH (85; 95% CI: 83, 86) compared to those with low social support (77; 95% CI: 76, 79) at year 1. Estimated SRH values declined to 53 (95% CI: 51, 55) and 46 (95% CI: 43, 49) for high social support and low social support respectively by year 28.

**Fig 2:**
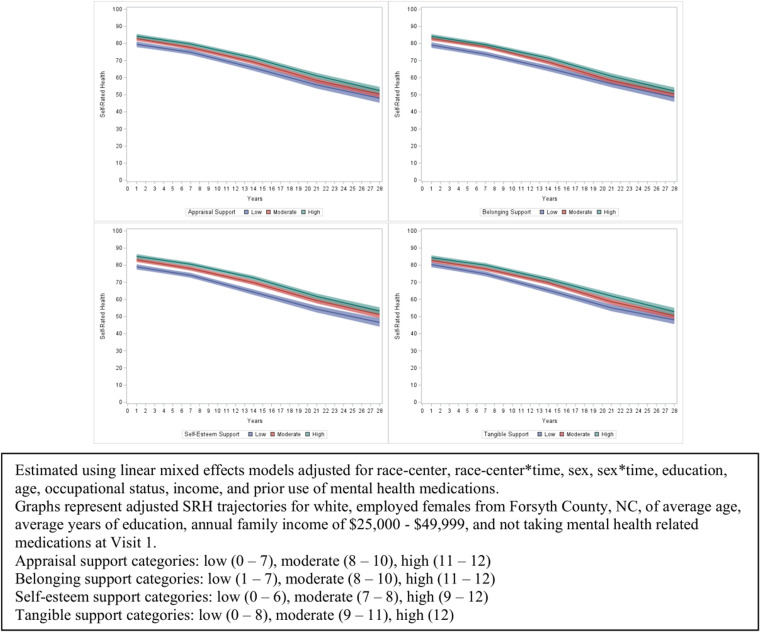
Associations of appraisal support, belonging support, self-esteem support, and tangible support with 28-year trajectories of self-rated health among Black and White middle-aged Americans in the Atherosclerosis Risk in Communities (ARIC) Study, with follow-up beginning 1990-1992.

SRH trajectories were similar for all types of social support; higher levels of each type of social support were associated with greater SRH over time compared to lower support of the same type (Fig 2). Although statistical tests suggested a differential rate of change in SRH over time by level of social support type, the rates of decline in SRH over time did not vary notably by level of social support type based on visual inspection of associations; this may be due to the large amount of data. The greatest difference in estimated SRH over follow-up when comparing those with high support to low support was for self-esteem support, and the smallest difference in predicted SRH by levels of support was for tangible support.

### Effect measure modification (ARIC)

Likelihood ratio test p-values indicated statistical differences in the associations between social relationship measures and SRH over time by sex, race, and marital status (see p-values in footnotes of S1-S3 Figs). This may be due to the large amount of data and therefore we based effect measure modification conclusions predominantly on visual inspection of associations. The association of social isolation with longitudinal SRH was slightly greater among females compared to males, but associations of social support were similar by sex (S1 Fig).

Differential rates of decline in SRH over time by baseline social isolation were observed only among White participants; social isolation was not associated with longitudinal SRH among Black participants (S2 Fig). The association between social support and longitudinal SRH was slightly greater among White participants compared to Black participants in later years of follow-up. However, confidence intervals for estimates of Black participants were wide, and it is possible that the widening difference in estimated SRH near the end of follow-up could reflect differential missingness in SRH over time by race. The association of social support with longitudinal SRH was greater among those who were not married compared to those who were married (S3 Fig). There were no differences in the association of social isolation with longitudinal SRH by marital status. The association between social support and SRH over time was not modified by social isolation (p = 0.24).

### Ten-year change in social relationships (ARIC/JHS)

On average (SD), the second social relationship measurement occurred 10.7 (0.6) years after baseline. The ARIC/JHS shared cohort included fewer married individuals (71%) and had a higher proportion of those classified as socially isolated at baseline (2.3%) (Table 1). This sample had slightly higher overall social support scores (median (25^th^, 75^th^ percentile): 39 (34, 43)) compared to the full ARIC sample at baseline.

Social isolation scores had a median (25^th^, 75^th^ percentile) 10-year change of −2 (−3, 0) and social support scores had a median (25^th^, 75^th^ percentile) 10-year change of 0 (−5, 4), indicating an increase in risk for social isolation and stable social support (S3 Table). Participants who experienced stable high risk for social isolation and stable low levels of social support over the 10-year period had fewer years of education (Table 2) and were more often unmarried at both time points (S4 Table). These participants tended to have poorer mental health-related variables at both baseline and the time of the second social relationship measurement. Participants who experienced stable low social support also had a greater burden of cardiovascular disease between social relationship measurements. In contrast, those who had stable high social support had more positive cardiovascular risk factors such as being non-smokers and engaging in physical activity, and had higher scores on cognitive functioning measures.

**Table 2:** Sociodemographic and clinical characteristics of the ARIC/JHS shared cohort at ARIC Visit 2 (1990-1992), by categories of 10-year changes in social isolation and social support; N = 911.

	N (%) or mean ± SD or median [25^th^ %, 75^th^ %]							
	Social isolation				Social support			
	Stable High N = 30 (3.3%)	Increase N = 212 (23.3%)	Decrease N = 24 (2.6%)	Stable Low or Moderate N = 645 (70.8%)	Stable Low N = 29 (3.2%)	Decrease N = 83 (9.1%)	Increase N = 63 (6.9%)	Stable High or Moderate N = 736 (80.8%)
**Demographic factors**								
Females	18 (60.0)	123 (58.0)	14 (58.3)	451 (69.9)	21 (72.4)	48 (57.8)	42 (66.7)	495 (67.3)
Education, years	13.9 ± 4.8	14.9 ± 5.1	14.2 ± 4.9	15.2 ± 5.0	12.9 ± 4.4	13.3 ± 4.9	13.4 ± 4.9	15.5 ± 5.0
Age, years	54.6 ± 5.5	55.6 ± 5.4	56.0 ± 6.4	54.8 ± 5.4	54.8 ± 5.5	56.2 ± 6.2	55.1 ± 5.5	54.9 ± 5.3
Employment status								
Homemaker	0 (0.0)	8 (3.9)	2 (8.3)	53 (8.2)	2 (6.9)	7 (8.5)	5 (8.1)	49 (6.7)
Employed	28 (93.3)	182 (87.5)	20 (83.3)	536 (83.4)	25 (86.2)	61 (74.4)	52 (83.9)	628 (85.8)
Unemployed	0 (0.0)	4 (1.9)	1 (4.2)	11 (1.7)	1 (3.5)	1 (1.2)	1 (1.6)	13 (1.8)
Retired	2 (6.7)	14 (6.7)	1 (4.2)	43 (6.9)	1 (3.5)	13 (15.8)	4 (6.5)	42 (5.7)
*Missing*	0	4	0	2	0	1	1	4
Health insurance	26 (86.7)	172 (81.1)	20 (83.3)	521 (80.8)	22 (75.9)	61 (73.5)	46 (73.0)	610 (82.9)
Household income								
Under $25,000	17 (63.0)	110 (58.2)	14 (73.7)	332 (58.0)	17 (68.0)	49 (67.1)	42 (70.0)	365 (56.2)
$25,000 - $49,999	7 (25.9)	63 (33.3)	4 (21.1)	178 (31.1)	8 (32.0)	23 (31.5)	12 (20.0)	209 (32.2)
Over $50,000	3 (11.1)	16 (8.5)	1 (5.3)	62 (10.8)	0 (0.0)	1 (1.4)	6 (10.0)	75 (11.6)
*Missing*	3	23	5	73	4	10	3	87
Married	16 (59.3)	142 (71.4)	16 (72.7)	426 (71.5)	13 (48.2)	51 (68.9)	37 (69.8)	499 (72.3)
Living arrangement								
With spouse	15 (50.0)	137 (65.9)	15 (62.5)	402 (62.8)	10 (34.5)	51 (62.2)	35 (55.6)	473 (65.0)
With others (not spouse)	11 (36.7)	42 (20.2)	4 (16.7)	165 (25.8)	13 (44.8)	18 (22.0)	21 (33.3)	170 (23.4)
Alone	4 (13.3)	29 (13.9)	5 (20.8)	73 (11.4)	6 (20.7)	13 (15.9)	7 (11.1)	85 (11.7)
**Mental health variables**								
Medication use* (Visit 1)	5 (16.7)	18 (8.5)	1 (4.2)	62 (9.6)	6 (20.7)	8 (9.6)	7 (11.1)	65 (8.8)
Medication use* (Visit 2)	6 (20.0)	18 (8.5)	1 (4.2)	53 (8.2)	7 (24.1)	9 (10.8)	8 (12.7)	54 (7.3)
Vital exhaustion score	15.0 ± 9.3	9.9 ± 8.2	14.3 ± 9.5	10.3 ± 8.0	17.7 ± 11.3	13.0 ± 8.3	13.7 ± 10.2	9.5 ± 7.5
**Clinical cardiovascular risk factors and diseases**								
Hypertension	15 (50.0)	77 (36.5)	9 (37.5)	259 (40.4)	14 (48.3)	38 (45.8)	31 (49.2)	277 (37.9)
Total cholesterol, mg/dL	209 ± 35	213 ± 39	202 ± 31	207 ± 41	207 ± 42	207 ± 39	209 ± 41	209 ± 40
LDL cholesterol, mg/dL	127 ± 35	137 ± 36	126 ± 29	132 ± 40	129 ± 39	131 ± 38	133 ± 36	133 ± 39
Cholesterol medication use	1 (3.3)	7 (3.3)	2 (8.3)	18 (2.8)	0 (0.0)	5 (6.0)	4 (6.4)	19 (2.6)
Diabetes	5 (16.7)	23 (11.0)	7 (30.4)	86 (13.5)	3 (10.3)	11 (13.4)	11 (17.7)	96 (13.2)
Body mass index, kg/m^2^	27.7 ± 5.6	29.44 ± 5.3	30.7 ± 5.2	29.7 ± 5.8	30.9 ± 6.3	29.7 ± 6.1	29.2 ± 6.1	29.6 ± 5.5
Cognitive function score								
Total words recalled	6.0 ± 1.5	6.4 ± 1.4	5.9 ± 1.4	6.6 ± 1.5	6.4 ± 1.6	6.2 ± 1.6	6.4 ± 1.3	6.5 ± 1.5
Total correct symbols	34.2 ± 14.5	34.2 ± 12.9	32.9 ± 13.9	36.1 ± 13.0	31.5 ± 10.9	32.5 ± 12.2	31.1 ± 13.3	36.4 ± 13.0
Total incorrect symbols	0.1 ± 0.4	0.5 ± 3.3	0.2 ± 0.5	0.2 ± 0.7	0.0 ± 0.0	0.2 ± 0.8	0.7 ± 2.4	0.2 ± 1.7
Total words listed	27.8 ± 14.4	30.2 ± 14.0	30.4 ± 16.0	32.2 ± 13.2	25.4 ± 13.5	27.0 ± 12.6	27.8 ± 13.9	32.6 ± 13.3
Self-rated health								
Excellent	4 (13.3)	57 (26.9)	3 (13.0)	136 (21.1)	3 (10.3)	11 (13.3)	12 (19.4)	174 (23.7)
Good	14 (46.7)	114 (53.8)	13 (56.5)	364 (56.5)	15 (51.7)	47 (56.6)	34 (54.8)	409 (55.7)
Fair	8 (26.7)	38 (17.9)	5 (21.7)	134 (20.8)	8 (27.6)	22 (26.5)	15 (24.2)	140 (19.1)
Poor	4 (13.3)	3 (1.4)	2 (8.7)	10 (1.6)	3 (10.3)	3 (3.6)	1 (1.6)	12 (1.6)
**Health behaviors**								
Mins/week of MVPA	0 [0, 60]	0 [0, 113]	39 [0, 282]	0 [0, 126]	0 [0, 0]	0 [0, 181]	0 [0, 141]	0 [0, 123]
Diet								
Daily fruit servings	0.9 [0.4, 2.1]	1.5 [0.8, 2.1]	2.0 [1.4, 3.0]	1.6 [0.8, 2.6]	1.4 [0.4, 2.0]	1.4 [0.7, 2.4]	1.6 [0.7, 2.2]	0.6 [0.8, 2.5]
Daily vegetable servings	0.6 [0.4, 0.8]	0.6 [0.4, 1.0]	1.0 [0.5, 1.4]	0.6 [0.4, 1.1]	0.4 [0.3, 0.8]	0.6 [0.4, 1.0]	0.6 [0.4, 1.0]	0.6 [0.4, 1.1]
Daily fish servings	0.4 [0.2, 0.6]	0.3 [0.2, 0.6]	0.3 [0.2, 0.6]	0.3 [0.2, 0.6]	0.2 [0.1, 0.4]	0.3 [0.2, 0.6]	0.3 [0.2, 0.6]	0.3 [0.2, 0.6]
Smoking status								
Current	6 (20.0)	45 (21.2)	4 (16.7)	126 (19.6)	6 (20.7)	23 (27.7)	14 (22.2)	138 (18.8)
Former	12 (40.0)	61 (28.8)	12 (50.0)	186 (28.9)	9 (31.0)	26 (31.3)	19 (30.2)	217 (29.6)
Never	12 (40.0)	106 (50.0)	8 (33.3)	331 (51.5)	14 (48.3)	34 (41.0)	30 (47.6)	379 (51.6)
Alcohol use								
Current	10 (33.3)	81 (38.2)	5 (20.8)	226 (35.0)	13 (44.8)	29 (34.9)	19 (30.2)	261 (35.5)
Former	8 (26.7)	59 (27.8)	9 (37.5)	168 (26.1)	5 (17.2)	24 (28.9)	26 (41.3)	189 (25.7)
Never	12 (40.0)	72 (34.0)	10 (41.7)	250 (38.8)	11 (37.9)	30 (36.1)	18 (28.6)	285 (38.7)
Frequency of physical examinations								
No routine examinations	8 (26.7)	41 (19.3)	5 (20.8)	109 (16.9)	7 (24.1)	20 (24.1)	13 (20.6)	123 (16.7)
≥ every 5 years	5 (16.7)	30 (14.2)	2 (8.3)	95 (14.7)	2 (6.9)	9 (10.8)	11 (17.5)	110 (15.0)
<every 5 years	0 (0.0)	4 (1.9)	0 (0.0)	13 (2.0)	0 (0.0)	1 (1.2)	1 (1.6)	15 (2.0)
≥ once a year	17 (56.7)	137 (64.6)	17 (70.8)	428 (66.4)	20 (69.0)	53 (63.9)	38 (60.3)	488 (66.3)

N: number; SD: standard deviation; Mg: milligram; dL: deciliter; kg: kilograms; m: meter; MVPA: moderate-to-vigorous physical activity

*Mental health medications include those indicated for depression and anxiety.

**Fig 3:**
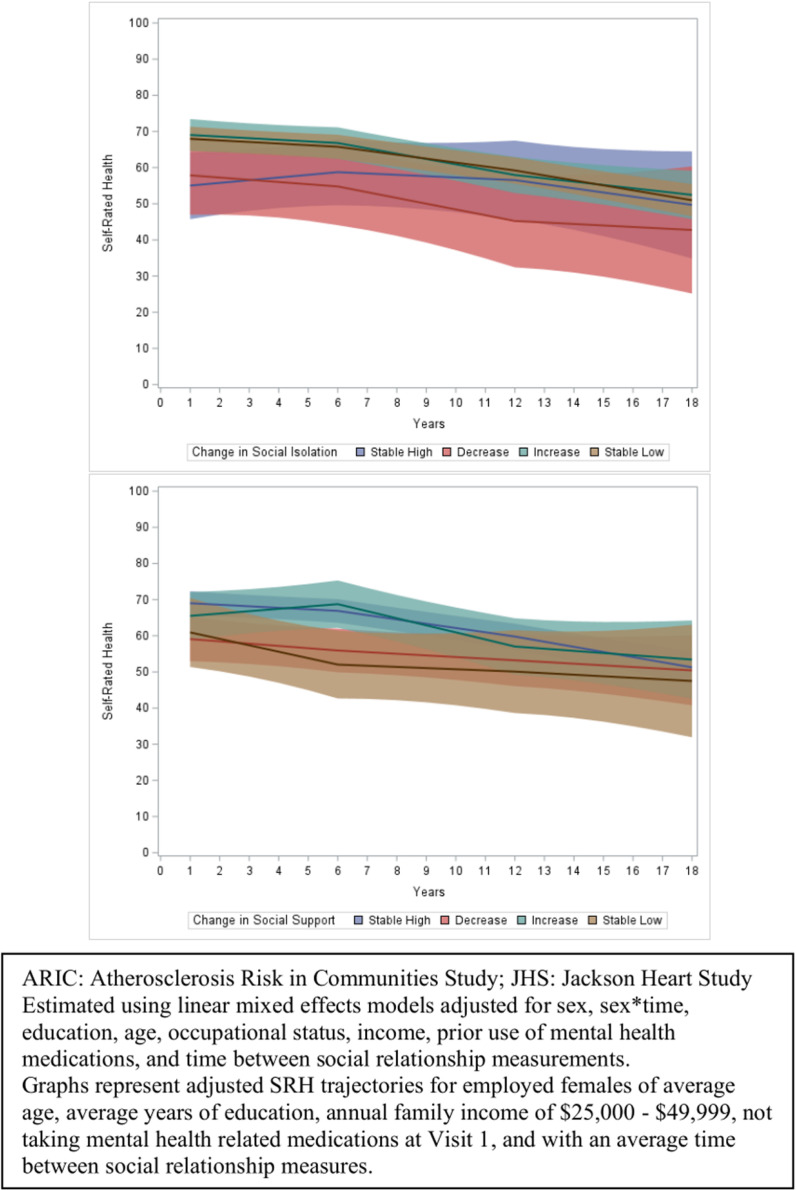
Associations of categories of 10-year changes in social isolation and social support with 18-year trajectories of self-rated health among Black middle-aged Americans in the ARIC/JHS shared cohort, with first social isolation/support measurement occurring in 1990-1992.

Although confidence intervals were wide, overall SRH overtime varied by level of change in social relationship variables, but the rate of decline in SRH did not appear to be differential by changes in social relationship variables (Fig 3). Estimated longitudinal SRH was highest among those with stable low risk for social isolation compared to those who experienced a decrease in social isolation or those with stable high risk for social isolation. Additionally, estimated longitudinal SRH was highest for stable high social support and increases in social support, compared to decreases in social support and stable low social support. These same patterns were present when assessing different types of social support (S4 Fig).

### Sensitivity analyses

Further adjustment for baseline variables that may be related to SRH non-response (sensitivity analysis 1), and the addition of participants with prevalent cardiovascular disease and cancer at baseline (sensitivity analysis 2) did not alter inferences made from main results. When the association between social support and SRH over time was adjusted for social isolation, the magnitude of association was similar but confidence intervals were slightly wider (sensitivity analysis 3).

All associations were attenuated after adjustment for baseline use of mental health-related medications and vital exhaustion scores (sensitivity analysis 4). The association between risk for social isolation and estimated SRH over time was present only among those who were married, while low social support was associated with poorer estimated SRH over time only among those who were not married. For analyses of changes in social relationships, only a decrease in social isolation was associated with lower estimated SRH over time.

When using the alternative dichotomous scoring system to assess changes in risk for social isolation, estimated SRH probability trajectories did not vary much by 10-year change in risk for social isolation (sensitivity analysis 5). SRH over time was slightly higher for those with stable high risk for social isolation and those with an increase in social isolation, and SRH was slightly lower over time for those with a decrease in and stable low risk for social isolation, although confidence intervals were wide.

## Discussion

In this cohort of middle-aged Black and White adults followed for 28 years, we found that low risk for social isolation and high social support were associated with better SRH over time. The rate of decline in SRH was slightly faster for those with high risk for social isolation compared to those with low risk for social isolation, but the rate of decline in SRH was not differential by levels of social support. The association between social isolation and longitudinal SRH was slightly stronger among females compared to males, and was present only among White individuals and not Black individuals, but did not differ by marital status. The association between social support and longitudinal SRH was slightly greater among White individuals compared to Black individuals, and individuals who were not married compared to those who were, but did not differ by sex. Overall, social relationship measures were relatively stable over ten years; those with high social support or low risk for social isolation across 10-years may have greater estimated SRH over time.

Social relationships may impact health by altering behaviors (i.e., smoking, medication adherence, diet, and physical activity) and psychological well-being (i.e., stress management and depression), which may in turn impact risk of disease morbidity and mortality [39]. The observed attenuation of the associations of social isolation and social support with SRH following adjustment for vital exhaustion and mental health-related medications at the time of social relationship measurement lends support for the latter hypothesis. However, our findings do not necessarily negate a potential pathway through health-related behaviors as we did not evaluate health-related behaviors in this study. Social relationship measures may also be impacted by disease, as greater need for social support may increase with declining health status and declining health status may strain existing relationships [21]. However, we did not observe any differences in results when excluding participants with prevalent chronic diseases compared to results which included these participants.

Additionally, different aspects of social relationships and social support may impact health through various mechanisms such as 1) sharing health-related information (appraisal support) [30], 2) using money or other gifts to improve health or quality of life (tangible support) [1], 3) receiving help with household chores, daily living errands, and transportation to and from healthcare appointments (tangible support) [1], and 4) by creating social stability which may improve mental health and immune function (belonging and self-esteem support, and low social isolation) [30]. Our study found that self-esteem support had the greatest magnitude of association with SRH over time and that tangible support had the smallest magnitude of association, suggesting that future interventions may focus on increasing self-esteem support.

We are aware of only four studies that examined baseline measures of social relationships and their associations with longitudinal SRH, with follow-up times ranging from 2 to 19 years, and the number of SRH assessments ranging from 5 to 7 [40–43]. Two of these studies that used measures similar to social isolation found results in agreement with ours [41,42]. However, previous studies that used measures similar to social support did not find evidence of an association with longitudinal changes in SRH, unlike our results [40,43]. Lack of an association in previous studies may be due to short periods of time over which SRH trajectories were assessed and little to no decline in SRH over this time, with one study evaluating 4-year trajectories of SRH [40] and the other assessing 2-year trajectories [43]. Other reasons for conflicting results may include the use of different social relationship measurement tools (many of which were not validated), different analysis populations (African Americans only [40], Japanese adults [42], and retired/older Americans [41,43]), and the lack of incorporation of deaths into SRH values.

We found differences in associations between social relationship measures and longitudinal SRH by sex, race, and marital status. These differences may be due to differences in physical or mental health status associated with social relationship measures, or may be reflective of established variation of social relationships by these factors. For instance, prior studies found that females tended to have a greater number of social contacts and may benefit more from having a greater number and frequency of social interactions, while males may benefit more from specific aspects of social support such as tangible support [44,45]. While there have been hypothesized differences in social relationships by race due to factors such as intergenerational family structure and living arrangements, collectivism worldviews, and religiosity, prior studies disagree whether White or Black Americans have greater number of social connections and greater social support [44,46]. Finally, marital status is often used as a proxy for social support and is one of many constructs on some social support questionnaires. While marital status was not incorporated in the social relationship questionnaires used in this study, having a partner to provide social support may explain differences in the associations by this factor. As evidence of differences in social relationships by these factors, a previous ARIC study showed that there was a higher correlation between the LSNS and the ISEL among White participants compared to their Black counterparts, but that study did not observe differences in this correlation by sex [34].

Previously reported differences in SRH by sex, race, and marital status may also partially explain these effect measure modification results. The types of health conditions considered and the implicit referent to which comparison is made when selecting a SRH category, predictive validity in terms of physical health measures, and reliability of SRH, have all been shown to vary by these factors [12]. Therefore, prior literature argues that SRH may not be the ideal measure for identifying physical or mental health disparities across groups, and that researchers should be cautious when labeling differences in SRH across these groups as clinical health disparities [47]. Although differences found by sex, race, and marital status cannot be interpreted as disparities in physical or mental health status, results may point to social relationship factors that are more predictive of physical and mental health conditions among certain groups.

Even though social relationships are hypothesized to change surrounding life events [15,16], our study found that social support measured at mid-life and then 10 years later was relatively stable, and that there was a slight increase in social isolation. While social relationships may change surrounding life events, the socioemotional theory suggests that as people age, they prune their social connections in order to maximize social support provided by their contacts [45]. We found that participants who maintained low risk for social isolation or high social support at both time points tended to be in better physical and mental health at both time points, had higher socio-economic characteristics, and exhibited more favorable health behaviors. This information may help in the identification of those who may benefit most from social relationship interventions. Finally, our results suggested that maintaining low risk for social isolation and high social support over the course of 10 years may be associated with better subsequent longitudinal SRH. Other previous studies have found that maintaining positive social relationship measures [48,49] or improving social relationship measures [50,51] over time may be associated with better SRH, although not all results agree [52,53].

### Strengths and limitations

A strength of this study was the specification of the SRH variable that included death, the most extreme health outcome. This reduced survivor bias compared to that present in the common practice of excluding deaths from analysis, as those who died during follow-up would have had missing SRH values. Another strength of this study is the length of prospective follow-up, and the number of assessments and frequency of SRH measurements. Additionally, few prior studies have investigated both social isolation and social support in relation to longitudinal SRH in the same population, and even fewer assessed how different types of social support are associated with SRH over time. It may be particularly important to assess both social isolation (number of connections) and social support (perception of support adequacy) in the same study as personality factors may influence an individual’s social needs, which may be reflected in social support measures but not social isolation measures. We also utilized widely-recognized and valid multi-question measures of social isolation and social support, while other previous studies predominantly did not.

We were limited in that we were unable to assess potential negative social interactions; although the lack of social isolation typically has a positive connotation, the existence of social support may still include negative social interactions. Furthermore, the prevalence of social isolation was relatively low in this population, and social support scores were also fairly high. This may be due to differences in characteristics between cohort study participants and the general population, who tend to be healthier than the average person. Additionally, participants that were excluded due to missing social relationship measures tended to be in poorer physical and mental health, and had lower socioeconomic status measures compared to those with complete measures. These characteristics were similar to participants with high risk for social isolation and who had low social support. Therefore, our results may be an underestimate of the true association in the general population based on social relationship measure missingness and low overall prevalence of social isolation and low social support.

It is possible that high social support may contribute positively to one’s perception of health, leading to better SRH. In fact, some studies have shown that subjects often consider their social well-being as one of many factors when reporting SRH [30,54]; this may have been part of the reason why we observed an association. The assessment of changes in social relationships was limited by lower statistical power, and by measurement only among Black participants, reducing the generalizability of these results. Additionally, results from the sensitivity analysis using a different scoring method for assessment of changes in social isolation suggested that this association was sensitive regarding the scoring method used to compare social isolation measures. This may indicate that the LSNS and BSNI are not easily compared across time points, and that multiple measurements of social isolation using the same questionnaire over time may be better for future works.

## Conclusions

Low risk for social isolation and high social support were both associated with greater perceived health over time. Of the different types of social support, self-esteem support had the greatest magnitude association with longitudinal SRH. Over a ten-year period, social support remained fairly stable while social isolation increased slightly. Results of our study suggest that interventions aimed to improve health among middle-aged adults may benefit from reducing social isolation and increasing social support, particularly self-esteem support, among certain subgroups.

## Supporting information

S1 Table Questions and scoring used to assess changes in structural aspects of social relationships using questions from the Lubben Social Network Score (LSNS) and the Berkman-Syme Social Network Index (BSNI). (DOCX)

S2 Table Sociodemographic and clinical characteristics of the ARIC cohort and Visit 2 (1990–1992), stratified by missing status of self-rated health variable at year 28 of follow-up. (DOCX)

S3 Table Descriptive statistics of social relationship variables at ARIC Visit 2 (1990–1992) and 10 years later at JHS Visit 1 (2000–2005) among ARIC/JHS shared cohort participants, N = 911. (DOCX)

S4 Table Sociodemographic and clinical characteristics of the ARIC/JHS shared cohort at JHS Visit 1 (2000–2004), by categories of 10-year changes in social isolation and social support; N = 911. (DOCX)

S1 Fig Associations of social isolation and social support with 28-year trajectories of self-rated health by sex in ARIC. (DOCX)

S2 Fig Associations of social isolation and social support with 28-year trajectories of self-rated health by race in ARIC. (DOCX)

S3 Fig Associations of social isolation and social support with 28-year trajectories of self-rated health by marital status in ARIC. (DOCX)

S4 Fig Associations of categories of 10-year changes in appraisal support, belonging support, self-esteem support, and tangible support with 18-year trajectories of self-rated health in the ARIC/JHS shared cohort. (DOCX)
