# Supplementary material for: Longitudinal, prospective cohort study of social relationships and self-rated health in the Atherosclerosis Risk in Communities (ARIC) Study cohort and ARIC/Jackson Heart Study (JHS) shared cohort
Source: PLoS One. 2025 Jun 13;20(6):e0326196. doi: 10.1371/journal.pone.0326196 (PMC12165402; doi:10.1371/journal.pone.0326196)
Supplement: S1 Table — (DOCX) [file pone.0326196.s001.docx]

| **S1 Table.** Questions and scoring used to assess changes in structural aspects of social relationships using questions from the Lubben Social Network Score (LSNS) and the Berkman-Syme Social Network Index (BSNI). | | | |
| --- | --- | --- | --- |
| LSNS Questions | | BSNI Questions | |
| Question Prompt | Response (Scoring) | Question Prompt | Response (Scoring) |
| 1.How many relatives do you feel close to? That is, how many of them do you feel at ease with, can talk to about private matters, or can call on for help? | Zero (0)  One (1)  Two (1)  Three or Four (2)  Five to Eight (3)  Nine or more (4) | 1.How many relatives do you have that you feel close to? | None (0)  1 or 2 (1)  3 to 5 (2)  6 to 9 (3)  10 or more (4) |
| 2.Do you have any close friends? That is, do you have any friends with whom you feel at ease, can talk to about private matters, or can call on for help? If so, how many? | Zero (0)  One (1)  Two (1)  Three or Four (2)  Five to Eight (3)  Nine or more (4) | 2.How many close friends do you have (people you feel at ease with, can talk to about private matters, and can call on for help)? | None (0)  1 or 2 (1)  3 to 5 (2)  6 to 9 (3)  10 or more (4) |
|  | | | |
| 3a.How many relatives do you see or hear from at least once a month?* | Zero (0)  One (1)  Two (2)  Three or Four (3.5)  Five to Eight (6.5)  Nine or more (9) | 3.How many of these friends or relatives do you see at least once per month? | None (0)  1 or 2 (1)  3 to 5 (2)  6 to 9 (3)  10 or more (4) |
| 3b.How many of these friends do you see or hear from at least once a month?* | Zero (0)  One (1)  Two (2)  Three or Four (3.5)  Five to Eight (6.5)  Nine or more (9) |  |  |
| *Scores from these two questions were summed together, and grouped according to the following categories for a final summary score of how many relatives and friends are seen each month:  0 = 0  1-2 = 1  3-4 = 2  5-8=3  9+=4 | |  | |
