# Supplementary material for: Longitudinal, prospective cohort study of social relationships and self-rated health in the Atherosclerosis Risk in Communities (ARIC) Study cohort and ARIC/Jackson Heart Study (JHS) shared cohort
Source: PLoS One. 2025 Jun 13;20(6):e0326196. doi: 10.1371/journal.pone.0326196 (PMC12165402; doi:10.1371/journal.pone.0326196)
Supplement: S2 Table — (DOCX) [file pone.0326196.s002.docx]

| **S2 Table.** Sociodemographic and clinical characteristics of the ARIC cohort and Visit 2 (1990-1992), stratified by missing status of self-rated health variable at year 28 of follow-up | | |
| --- | --- | --- |
|  | N (%) or mean ± SD or median [25^th^ %, 75^th^ %] | |
|  | Non-Missing  N=7967 | Missing  N=2888 |
| ARIC field center |  |  |
| Forsyth County, NC | 2075 (26.0) | 828 (28.7) |
| Jackson, MS | 1539 (19.3) | 804 (27.8) |
| Minneapolis, MN | 2377 (29.8) | 660 (22.9) |
| Washington County, MD | 1976 (24.8) | 596 (20.6) |
| Age, years | 56.5 ± 5.7 | 56.8 ± 5.6 |
| Females | 4415 (55.4) | 1729 (59.9) |
| Black participants | 1728 (21.7) | 896 (31.0) |
| Education, years | 14.7 ± 4.3 | 14.3 ± 4.3 |
| Employment status |  |  |
| Homemaker | 903 (11.4) | 371 (12.9) |
| Employed | 5826 (73.3) | 2074 (71.9) |
| Unemployed | 167 (2.1) | 58 (2.0) |
| Retired | 1053 (13.3) | 381 (13.2) |
| *Missing* | 18 | 4 |
| Household income |  |  |
| Under $25,000 | 2380 (31.7) | 982 (36.7) |
| $25,000 - $49,999 | 2951 (39.3) | 1049 (39.2) |
| Over $50,000 | 2187 (29.1) | 644 (24.1) |
| *Missing* | 449 | 213 |
| Married | 6419 (82.7) | 2308 (82.3) |
| Social isolation |  |  |
| Isolated (8 – 20) | 96 (1.2) | 38 (1.3) |
| High risk for isolation (21 – 25) | 341 (4.3) | 123 (4.3) |
| Moderate risk for isolation (26 – 30) | 1060 (13.3) | 387 (13.4) |
| Low risk for isolation (31 – 50) | 6470 (81.2) | 2340 (81.0) |
| Social support* |  |  |
| Overall | 38 [33, 42] | 38 [33, 42] |
| Appraisal social support | 10 [8, 11] | 10 [8, 11] |
| Belonging social support | 10 [8, 11] | 10 [8, 11] |
| Self-esteem social support | 8 [7, 9] | 8 [7, 10] |
| Tangible social support | 11 [9, 12] | 11 [9, 12] |
| Depression/anxiety medication use (Visit 1) | 668 (8.4) | 265 (9.2) |
| Depression/anxiety medication use (Visit 2) | 667 (8.4) | 296 (10.3) |
| Vital exhaustion score | 9.5 ± 8.2 | 9.9 ± 8.1 |
| Hypertension | 2056 (25.9) | 803 (27.9) |
| Total cholesterol, mg/dL | 208 ± 39 | 212 ± 39 |
| LDL cholesterol, mg/dL | 132 ± 37 | 136 ± 37 |
| High cholesterol medication use | 410 (5.2) | 140 (4.9) |
| Diabetes | 799 (10.1) | 260 (9.1) |
| Body mass index, kg/m^2^ | 27.7 ± 5.3 | 28.1 ± 5.2 |
| ARIC: Atherosclerosis Risk in Communities Study; N: number; SD: standard deviation; mg: milligrams; dL: deciliter; kg: kilograms; m: meter  *Social support variables presented as median [IQR] rather than mean (SD) due to skewed distribution | | |
