# Supplementary material for: Longitudinal, prospective cohort study of social relationships and self-rated health in the Atherosclerosis Risk in Communities (ARIC) Study cohort and ARIC/Jackson Heart Study (JHS) shared cohort
Source: PLoS One. 2025 Jun 13;20(6):e0326196. doi: 10.1371/journal.pone.0326196 (PMC12165402; doi:10.1371/journal.pone.0326196)
Supplement: S3 Table — (DOCX) [file pone.0326196.s003.docx]

| **S3 Table.** Descriptive statistics of social relationship variables at ARIC Visit 2 (1990-1992) and 10 years later at JHS Visit 1 (2000-2005) among ARIC/JHS shared cohort participants, N=911 | | | |
| --- | --- | --- | --- |
|  | Median [25^th^ %, 75^th^ %] | | |
|  | ARIC Visit 2  (1990-1992) | JHS Visit 1  (2000-2005) | 10-Year Change |
| Social isolation | 8 [6, 9] | 6 [4, 8] | -2 [-3, 0] |
| Social support |  |  |  |
| Overall | 39 [34, 43] | 39 [33, 43] | 0 [-5, 4] |
| Appraisal social support | 10 [9, 12] | 10 [8, 12] | 0 [-2, 1] |
| Belonging social support | 10 [8, 11] | 10 [8, 12] | 0 [-1, 1] |
| Self-esteem social support | 9 [7, 10] | 9 [7, 10] | 0 [-1, 1] |
| Tangible social support | 11 [9, 12] | 10 [8, 12] | 0 [-2, 1] |
| ARIC: Atherosclerosis Risk in Communities Study; JHS: Jackson Heart Study | | | |
