# Supplementary material for: Longitudinal, prospective cohort study of social relationships and self-rated health in the Atherosclerosis Risk in Communities (ARIC) Study cohort and ARIC/Jackson Heart Study (JHS) shared cohort
Source: PLoS One. 2025 Jun 13;20(6):e0326196. doi: 10.1371/journal.pone.0326196 (PMC12165402; doi:10.1371/journal.pone.0326196)
Supplement: S4 Table — (DOCX) [file pone.0326196.s004.docx]

| **S4 Table.** Sociodemographic and clinical characteristics of the ARIC/JHS shared cohort at JHS Visit 1 (2000-2004), by categories of 10-year changes in social isolation and social support; N=911 | | | | | | | | |
| --- | --- | --- | --- | --- | --- | --- | --- | --- |
|  | N (%) or mean ± SD or median [25^th^ %, 75^th^ %] | | | | | | | |
|  | Social isolation | | | | Social support | | | |
|  | Stable High  N=30  (3.3%) | Increase  N=212  (23.3%) | Decrease  N=24  (2.6%) | Stable Low or Moderate  N=645  (70.8%) | Stable Low  N=29  (3.2%) | Decrease  N=83  (9.1%) | Increase  N=63  (6.9%) | Stable High or Moderate  N=736  (80.8%) |
| **Demographic factors** |  |  |  |  |  |  |  |  |
| Health insurance | 28 (93.3) | 191 (90.5) | 22 (91.7) | 594 (92.4) | 25 (86.2) | 74 (89.2) | 58 (92.1) | 678 (92.5) |
| Change in marital status |  |  |  |  |  |  |  |  |
| Remained married | 15 (55.6) | 119 (59.8) | 10 (45.5) | 326 (54.8) | 10 (37.0) | 44 (59.5) | 31 (58.5) | 385 (55.9) |
| Remained not married | 11 (40.7) | 51 (25.6) | 6 (27.3) | 149 (25.0) | 12 (44.4) | 22 (29.7) | 16 (30.2) | 167 (24.2) |
| Married to not married | 1 (3.7) | 23 (11.6) | 6 (27.3) | 99 (16.6) | 3 (11.1) | 7 (9.5) | 6 (11.3) | 113 (16.4) |
| Not married to married | 0 (0.0) | 6 (3.0) | 0 (0.0) | 21 (3.5) | 2 (7.4) | 1 (1.4) | 0 (0.0) | 24 (3.5) |
| *Missing* | 3 | 13 | 2 | 50 | 2 | 9 | 10 | 47 |
| **Mental health variables** |  |  |  |  |  |  |  |  |
| Depressive symptoms | 13.5 ± 11.8 | 9.9 ± 6.7 | 10.7 ± 4.8 | 9.4 ± 6.3 | 17.7 ± 13.1 | 13.1 ± 7.8 | 11.6 ± 7.5 | 8.9 ± 5.8 |
| Discrimination burden | 2.4 ± 0.9 | 2.3 ± 0.8 | 2.6 ± 0.9 | 2.4 ± 0.8 | 2.2 ± 0.9 | 2.4 ± 0.8 | 2.4 ± 0.8 | 2.4 ± 0.8 |
| Perceived stress | 5.5 ± 4.3 | 3.4 ± 3.8 | 3.3 ± 4.0 | 3.6 ± 3.6 | 5.2 ± 3.8 | 4.5 ± 4.2 | 3.9 ± 3.8 | 3.5 ± 3.6 |
| **Clinical cardiovascular risk factors and diseases** |  |  |  |  |  |  |  |  |
| Hypertension | 19 (63.3) | 148 (69.8) | 19 (79.2) | 468 (72.6) | 24 (82.8) | 62 (74.7) | 47 (74.6) | 521 (70.8) |
| Total cholesterol, mg/dL | 201 ± 41 | 208 ± 45 | 197 ± 40 | 205 ± 42 | 199 ± 45 | 206 ± 41 | 202 ± 44 | 206 ± 42 |
| LDL cholesterol, mg/dL | 122 ± 35 | 130 ± 37 | 116 ± 34 | 129 ± 37 | 123 ± 35 | 129 ± 39 | 129 ± 38 | 129 ± 37 |
| Cholesterol medication use | 8 (26.7) | 43 (20.5) | 7 (29.2) | 132 (20.6) | 6 (20.7) | 15 (18.3) | 14 (22.2) | 155 (21.2) |
| Diabetes | 9 (30.0) | 59 (28.0) | 13 (56.5) | 188 (29.2) | 10 (34.5) | 22 (26.8) | 20 (31.8) | 217 (29.6) |
| Body mass index, kg/m^2^ | 29.3 ± 7.8 | 30.6 ± 5.7 | 31.7 ± 6.0 | 31.1 ± 6.3 | 32.2 ± 6.6 | 31.0 ± 6.5 | 30.9 ± 7.3 | 30.9 ± 6.1 |
| Self-rated health |  |  |  |  |  |  |  |  |
| Excellent | 2 (6.7) | 23 (10.9) | 1 (4.2) | 91 (14.2) | 1 (3.5) | 6 (7.2) | 10 (15.9) | 100 (13.6) |
| Good | 11 (36.7) | 120 (56.6) | 10 (41.7) | 369 (57.4) | 14 (48.3) | 31 (37.4) | 29 (46.0) | 436 (59.4) |
| Fair | 12 (40.0) | 64 (30.2) | 11 (45.8) | 166 (25.8) | 14 (48.3) | 37 (44.6) | 20 (31.8) | 182 (24.8) |
| Poor | 5 (16.7) | 5 (2.4) | 2 (8.3) | 17 (2.6) | 0 (0.0) | 9 (10.8) | 4 (6.4) | 16 (2.2) |
| Myocardial infarction | 2 (6.7) | 10 (4.7) | 2 (8.3) | 34 (5.3) | 5 (17.2) | 4 (4.8) | 4 (6.4) | 35 (4.8) |
| Stroke | 1 (3.3) | 7 (3.3) | 4 (16.7) | 25 (3.9) | 2 (6.9) | 9 (10.8) | 2 (3.2) | 24 (3.3) |
| Coronary heart disease | 3 (10.0) | 15 (7.1) | 4 (16.7) | 52 (8.1) | 7 (24.1) | 4 (4.8) | 4 (6.4) | 59 (8.0) |
| Chronic kidney disease | 2 (6.7) | 11 (5.2) | 1 (4.2) | 23 (3.6) | 3 (10.3) | 5 (6.0) | 4 (6.4) | 25 (3.4) |
| **Health behaviors** |  |  |  |  |  |  |  |  |
| Physical activity (AHA categories) |  |  |  |  |  |  |  |  |
| Ideal | 1 (3.3) | 23 (10.9) | 1 (4.2) | 114 (17.7) | 2 (6.9) | 7 (8.4) | 9 (14.3) | 121 (16.4) |
| Intermediate | 13 (43.3) | 56 (26.4) | 6 (25.0) | 218 (33.8) | 8 (27.6) | 22 (26.5) | 18 (28.6) | 245 (33.3) |
| Poor | 16 (53.3) | 133 (62.7) | 17 (70.8) | 313 (48.5) | 19 (65.5) | 54 (65.1) | 36 (57.1) | 370 (50.3) |
| Diet |  |  |  |  |  |  |  |  |
| Daily vegetable servings | 0.2 [0.1, 0.5] | 0.2 [0.1, 0.4] | 0.3 [0.2, 0.4] | 0.3 [0.2, 0.4] | 0.3 [0.1, 0.4] | 0.2 [0.1, 0.4] | 0.3 [0.1, 0.4] | 0.3 [0.2, 0.4] |
| Daily fish servings | 0.1 [0.0, 0.1] | 0.1 [0.0, 0.2] | 0.1 [0.0, 0.3] | 0.1 [0.0, 0.2] | 0.2 [0.0, 0.2] | 0.1 [0.0, 0.2] | 0.1 [0.0, 0.2] | 0.1 [0.0, 0.2] |
| SD: standard deviation; Mg: milligram; dL: deciliter; kg: kilogram; m: meter; AHA: American Heart Association | | | | | | | | |
