# Supplementary material for: Longitudinal, prospective cohort study of social relationships and self-rated health in the Atherosclerosis Risk in Communities (ARIC) Study cohort and ARIC/Jackson Heart Study (JHS) shared cohort
Source: PLoS One. 2025 Jun 13;20(6):e0326196. doi: 10.1371/journal.pone.0326196 (PMC12165402; doi:10.1371/journal.pone.0326196)
Supplement: S1 Fig — (DOCX) [file pone.0326196.s005.docx]

**S1 Figure.** Associations of social isolation and social support with 28-year trajectories of self-rated health by sex in ARIC.


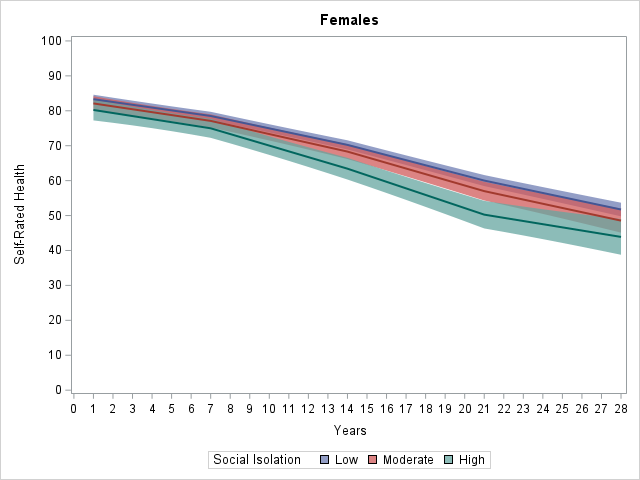

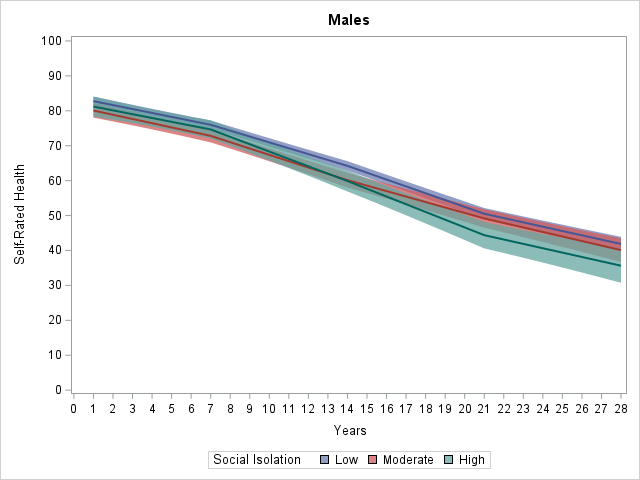


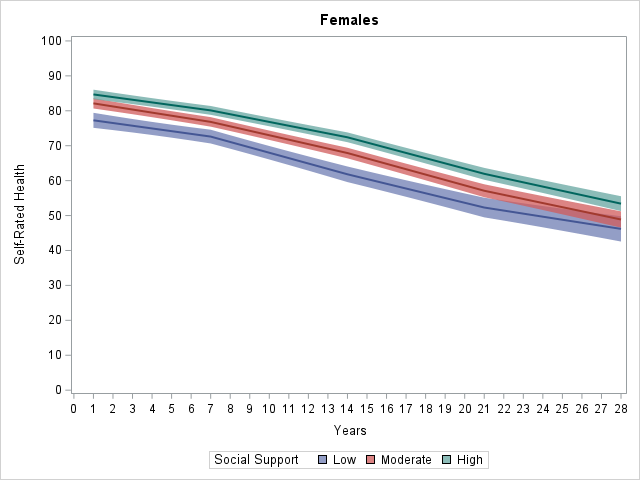

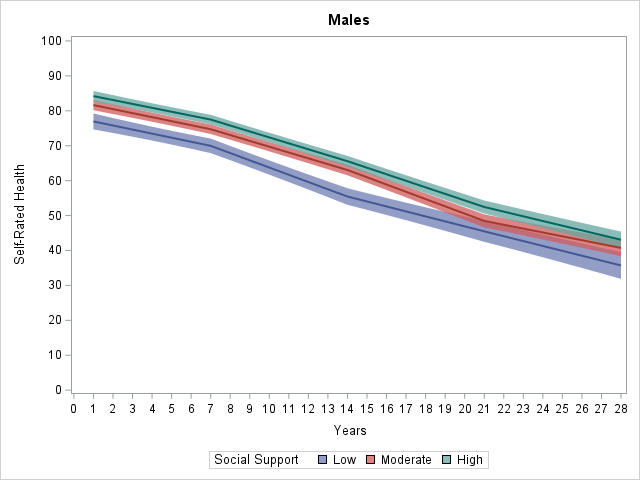


ARIC: Atherosclerosis Risk in Communities Study

Estimated using linear mixed effects models adjusted for race-center, race-center*time, education, age, occupational status, income, and prior use of mental health medications.

Graphs represent adjusted SRH trajectories for white, employed individuals from Forsyth County, NC, of average age, average years of education, annual family income of $25,000 - $49,999, and not taking mental health related medications at Visit 1.

Social isolation categories: socially isolated/high risk (8 – 25), moderate risk (26 – 30), low risk (31 – 50)

Social support categories: low (4 – 29), moderate (30 – 38), high (39 – 48)

P-value for interaction terms: social isolation*sex*time=0.008, social support*sex*time=0.040
