# Supplementary material for: Longitudinal, prospective cohort study of social relationships and self-rated health in the Atherosclerosis Risk in Communities (ARIC) Study cohort and ARIC/Jackson Heart Study (JHS) shared cohort
Source: PLoS One. 2025 Jun 13;20(6):e0326196. doi: 10.1371/journal.pone.0326196 (PMC12165402; doi:10.1371/journal.pone.0326196)
Supplement: S4 Fig — (DOCX) [file pone.0326196.s008.docx]

**S4 Figure.** Associations of categories of 10-year changes in appraisal support, belonging support, self-esteem support, and tangible support with 18-year trajectories of self-rated health in the ARIC/JHS shared cohort.


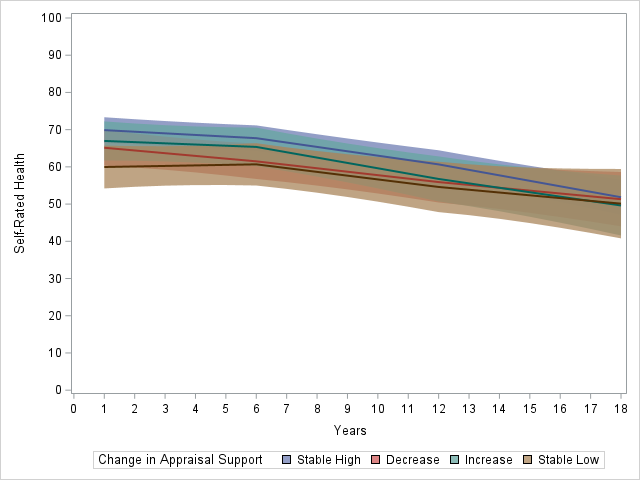

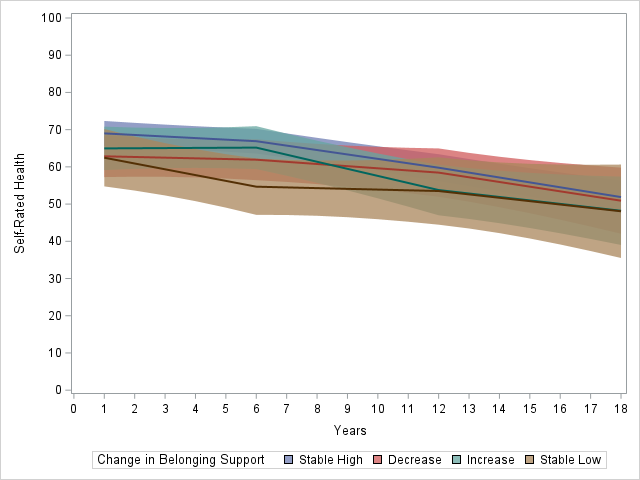


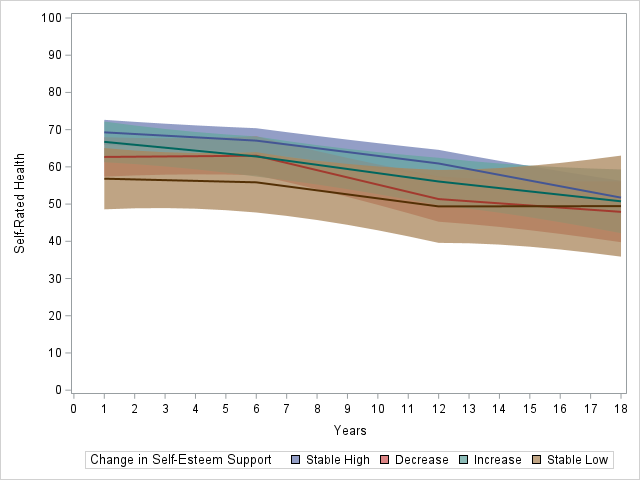

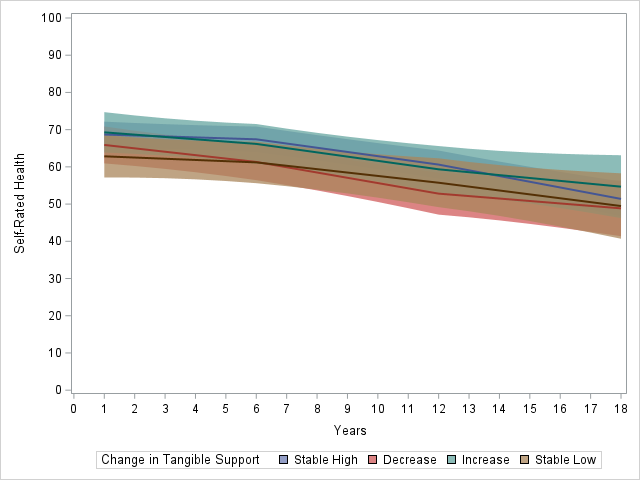


ARIC: Atherosclerosis Risk in Communities Study; JHS: Jackson Heart Study

Estimated using linear mixed effects models adjusted for sex, sex*time, education, age, occupational status, income, prior use of mental health medications, and time between social relationship measurements.

Graphs represent adjusted SRH trajectories for employed females of average age, average years of education, annual family income of $25,000 - $49,999, not taking mental health related medications at Visit 1, and with an average time between social relationship measures.
